# Dynamic changes in blood immune cell composition and function in Holstein and Jersey steers in response to heat stress

**DOI:** 10.1007/s12192-021-01216-2

**Published:** 2021-06-03

**Authors:** Da Som Park, Bon-Hee Gu, Yei Ju Park, Sang Seok Joo, Sang-Suk Lee, Seon-Ho Kim, Eun Tae Kim, Dong Hyeon Kim, Sung Sill Lee, Shin Ja Lee, Byeong-Woo Kim, Myunghoo Kim

**Affiliations:** 1 Department of Animal Science, College of Natural Resources & Life Science, Pusan National University, Miryang, 50463 Republic of Korea; 2 Life and Industry Convergence Research Institute, Pusan National University, Miryang, 50463 Republic of Korea; 3 Rumunant Nutrition and Anaerobe Laboratory, Department of Animal Science and Technology, Sunchon National University, Suncheon, 57922 Republic of Korea; 4 Dairy Science Division, National Institute of Animal Science, Rural Development Administration, Cheonan, 31000 Republic of Korea; 5 Division of Applied Life Science (BK21), Gyeongsang National University, Gyeongsangnam-do Jinju-si, 52828 Republic of Korea; 6 Institute of Agriculture and Life Science & University-Centered Labs, Gyeongsang National University, Gyeongsangnam-do Jinju-si, 52828 Republic of Korea

**Keywords:** Heat stress, Steer, Holstein, Jersey, Immunity, Flow cytometry

## Abstract

Heat stress has detrimental effects on livestock via diverse immune and physiological changes; heat-stressed animals are rendered susceptible to diverse diseases. However, there is relatively little information available regarding the altered immune responses of domestic animals in heat stress environments, particularly in cattle steers. This study aimed to determine the changes in the immune responses of Holstein and Jersey steers under heat stress. We assessed blood immune cells and their functions in the steers of two breeds under normal and heat stress conditions and found that immune cell proportions and functions were altered in response to different environmental conditions. Heat stress notably reduced the proportions of CD21^+^MHCII^+^ B cell populations in both breeds. We also observed breed-specific differences. Under heat stress, in Holstein steers, the expression of myeloperoxidase was reduced in the polymorphonuclear cells, whereas heat stress reduced the WC1^+^ γδ T cell populations in Jersey steers. Breed-specific changes were also detected based on gene expression. In response to heat stress, the expression of IL-10 and IL-17A increased in Holstein steers alone, whereas that of IL-6 increased in Jersey steers. Moreover, the mRNA expression pattern of heat shock protein genes such as Hsp70 and Hsp90 was significantly increased in only Holstein steers. Collectively, these results indicate that altered blood immunological profiles may provide a potential explanation for the enhanced susceptibility of heat-stressed steers to disease. The findings of this study provide important information that will contribute to developing new strategies to alleviate the detrimental effects of heat stress on steers.

## Introduction

Seasonal changes, particularly during the hot and humid season, can have detrimental effects on different aspects of livestock production, reproduction, metabolism, and immunity; recent trends in global warming seem exacerbate the adverse effects of environmental conditions during this season. For example, extreme weather conditions, such as the concurrence of heatwaves and droughts, tend to be associated with extremely high temperatures. Hot and humid environmental conditions induce heat stress responses in plants and animals, which are known to have significant effects on diverse physiological processes, including those related to metabolism and immunity, and can accordingly promote the development of diseases. In the USA, annual economic losses in the livestock industry, attributable to heat stress, have been estimated at $1.69 to $2.36 billion, with the dairy, beef, pig, and poultry industries accounting for 58%, 20%, 15%, and 7% of these losses, respectively (St-Pierre et al. 2003). Accordingly, it is imperative to gain a better understanding of heat stress physiology and develop strategies that can be implemented to alleviate the adverse effects of heat stress responses in livestock.

It has been reported that animals regularly subjected to heat stress are generally unhealthy and suffer from various diseases. The findings of correlation studies have indicated that heat-stressed animals have a higher incidence of diseases, including infectious and metabolism-related diseases. The increased incidence of diseases in heat-stressed animals can be attributed to impaired immune functions. Numerous studies have revealed the detrimental effects of heat stress on the immune status of livestock. For example, using RNAseq technology, it was found that Holstein bull calves exposed to severe heat stress show hyperinsulinemia, along with altered expression of genes involved in immune response and immunity-related signaling pathways (Srikanth et al. 2017). Relevant studies on the effect of heat stress have also demonstrated that there is an attenuated vaccine response in animals, which is characterized by abnormal immune responses (Bagath et al. 2019). Furthermore, the levels of immunoglobulin G2a (IgG2a), T cell proliferation, and the expression of interferon gamma (IFN-γ) in both CD4^+^ and CD8^+^ cells and cytotoxic T-lymphocyte have been reported to be suppressed under conditions of chronic heat stress (Hu et al. 2007). Consequently, administering vaccines to heat-stressed animals may not produce optimal immune responses, thereby reducing the efficacy of vaccination.

Levels of the hormone cortisol are among the most prominent biomarkers of heat stress responses in dairy cows. Cortisol production induced by heat stress stimulates the immune system, although conversely, the chronic secretion of cortisol is known to be associated with immune suppression (Ju et al. 2014; Jin et al. 2011). Several studies have assessed immunological changes in Holstein dairy cows under heat stress conditions. For example, the conditions associated with high temperature and dry environmental seasons have been found to affect immune responses, including reduction in lymphocyte proliferation, neutrophil phagocytosis, and cytokine expression in Holstein dairy cows (do Amaral et al. 2010, 2011). Moreover, heat-stressed Holstein cattle reportedly show reduced cellular immunity and enhanced humoral immune responses under prolonged heat stress (Lacetera et al. 2005). Several studies have reported that Jersey cows may be more heat tolerant than other breeds with respect to milk yield and fat percentage during severe heat stress (Harris et al. 1960; Collier et al. 1981; Smith et al. 2013). Some studies have also assessed the differential heat stress resistance among dairy cow breeds; it has been reported that Jersey cows can control the effects of thermal stress to a certain extent owing to their ability to dissipate internal heat via sweating (Knapp and Robinson 1954). However, although there have been a number of comparative studies on heat stress and physiological responses, the associated immunological properties have yet to be sufficiently investigated.

Dairy steers, particularly those of Holstein cows, are an important source of meat worldwide. Although dairy cows tend to be more sensitive to heat stress than steers, the latter are also prone to the adverse effects of unfavorable environmental conditions. For example, steers reared in breeding environments in which they are directly exposed to heat stress often show various clinical signs, including open-mouthed panting, drooling, reluctance, or inability to rise, increased licking of coats, and general dullness, and neurological signs, as manifested by staring glazed eyes. As indicated previously, economic losses in the beef industry due to heat stress can be considerable. For example, more than 4000 head of beef steers died in Iowa during a severe heat wave in 2006 (Belhadj Slimen et al., 2016). Heat stress has been shown to be associated with reduction in the body weight, average daily gain, and growth rate of beef cattle (Hahn 1999). Moreover, calves confined to hutches in summer with elevated body temperatures have been reported to have low levels of serum IgG and high mortality (Broucek et al. 2009). However, although heat stress has been shown to have a negative effect on the growth performance of steers, relatively few studies have examined changes in the productivity and physiology of steers subjected to heat stress.

Adverse environmental conditions also appear to influence the immune system of beef cattle; a few studies have examined the heat stress-related changes in immunological biomarkers in Holstein and Jersey steers. For example, it has been reported that a shift in the immune response from Th1 type (IFN-γ) to Th2 type (IL-4) in bulls during intense heat waves reflects a reduction in cell-mediated immunity (Peli et al. 2013).

Nevertheless, although heat stress clearly induces immunity-related changes in livestock animals, information pertaining to specific immune responses, including the response patterns of immune cells and their functions, tends to be limited, which can conceivably be attributed to technical limitations. For example, most relevant studies have only examined immune-associated biomarkers, such as cytokine levels, in dairy cows or steers subjected to different environmental stress conditions. As immune cells orchestrate responses in the immune system of animals, understanding the changes in these cells and their functions is essential for elucidating the related physiological mechanisms in animals. In the present study, we assessed blood immune cells and their functions in the steers of two cattle breeds (Holstein and Jersey) in normal (normal range of the temperature and humidity index (THI)) and heat stress (high THI condition) environments and attempted to identify common and breed-specific heat stress-sensitive immune cells and their functions. Our most interesting finding was that heat stress is associated with reduction in the proportions of CD21^+^MHCII^+^ B cells and cytotoxic T cells among blood immune-related cells. We believe that these changes in immune cells and functions may be associated with altered disease susceptibility in stressed steers.

## Materials and methods

### Animals

All experimental procedures for animal experiments were performed in accordance with the guidelines (SCNU-IACUC-2020-06) of the Institutional Animal Care and Use Committee (SCNU-IACUC), Republic of Korea. The principles of laboratory animal care were met, and blood collection procedures were performed by reducing pain and stress as much as possible, in accordance with the guidelines of the Korea National Standard of Cattle. All experimental protocols were approved by the Institutional Animal Care and Use Committee of the National Institute of Animal Science, Rural Development Administration, Republic of Korea. The six Holstein (690 ± 10.50 kg) and eight Jersey (550 ± 15.75 kg) steers used in this study were 36 months of age and housed in stalls. All cattle were fed selected diets once daily at 08:00 h, at a rate of 5–10% of the leftover diet.

### Calculation of the temperature humidity index

With respect to livestock, the THI is mainly used to reflect the intensity of heat stress; accordingly, in the present study, based on the available meteorological data, we used the THI to examine the effects of heat stress conditions on the aforementioned two representative dairy cow breeds. To gauge the load of heat stress on dairy cows, two distinct time slots were established as normal and high-temperature environments based on the THI for comparative experiments. For the determination of THI, we measured temperature and humidity using a thermos and humidity meter (Testo 174H Mini data logger; West Chester, PA, USA) and applied the following THI equation devised by the National Research Council (NRC, 1971): THI = (1.8 × ambient temperature + 32) - [(0.55 - 0.0055 × relative humidity) × (1.8 × ambient temperature - 26)]. For calculating the THI, measurements were conducted for normothermia from May 14^th^ to 27^th^ and for hyperthermia from August 5^th^ to 13^th^, for a total of 18 days; accordingly, we obtained average THI values of 64.92 and 79.13, respectively.

### Blood collection and immune cell isolation

Blood samples were obtained from the jugular vein of dairy cows and transferred to the Vacutainer tubes spray-coated with K2EDTA (BD Vacutainer; Becton Dickinson Co., Franklin Lakes, NJ, USA). The samples were immediately placed on ice and transported to the laboratory for the isolation of peripheral blood mononuclear cells (PBMCs) and polymorphonuclear cells (PMNs) within 30 min from the time of sampling. PBMCs were isolated by density-gradient centrifugation. Whole blood samples were diluted with phosphate-buffered saline (PBS) to a 1:1 ratio in 15-mL conical tubes. The diluted blood samples were then overlaid on the top of Lymphoprep (STEMCELL Technologies Inc., Vancouver, BC, Canada) in 15-mL conical tubes, and following centrifugation for 20 min at 800×*g* at room temperature (15–25°C), the layer of cells above the Lymphoprep was collected and washed twice with PBS to obtain purified dairy cow PBMCs. The isolation of PMNs was similar to that described for PBMCs, following density-gradient centrifugation. Similar to the PBMCs, the blood samples were diluted with PBS to a 1:1 ratio and then overlaid on the top of Lymphoprep. After centrifugation for 20 min at 800×*g* at room temperature, we observed two distinct layers in the blood. The whole layer beneath the PBMCs, the so-called interphase layer, was collected, and to this, we added red cell lysis buffer. After allowing it to settle for 15 min, the preparation was centrifuged for 5 min at 800×*g* at room temperature. The pellet thus obtained was washed with PBS, followed by further centrifugation for 3 min at 500*g* and room temperature to obtain highly purified PMNs. Samples for quantitative reverse transcription-PCR (qRT-PCR) were resuspended in 1 mL of the Trizol reagent (Invitrogen, CA, USA) and transferred to 1.5-mL tubes. PBMCs were immediately stored at −80°C until RNA isolation.

### Complete blood count analysis of the whole blood of dairy cows

All whole blood samples used for complete blood count (CBC) analysis were placed in 0.5-mL K3EDTA-coated tubes and immediately transported to the laboratory. Measurements were obtained using a Vetscan® HM5 hematological analyzer (ABAXIS, CA, USA), as recommended by the manufacturer, using settings for bovine blood, and confirmed in accordance with the manufacturer-recommended acceptable range prior to each series of analyses. Analyzer-measured variables included red blood cell (RBC) count; hemoglobin concentration (HGB); hematocrit; mean corpuscular volume; mean corpuscular hemoglobin; mean corpuscular hemoglobin concentration; red cell distribution width by standard deviation and coefficient of variation; RBC hemoglobin content (RBC-HGB); reticulocyte percentage (RET); immature reticulocyte fraction; low-, medium-, and high-fluorescence ratios as grades of reticulocyte maturation; reticulocyte hemoglobin content (RET-HGB); delta-HGB (calculated as the difference between reticulocyte and RBC hemoglobin content or RET-HGB minus RBC-HGB); WBC count; neutrophil, lymphocyte, monocyte, eosinophil, and basophil counts; platelet count by impedance and optical measurements; mean platelet volume; platelet distribution width; plateletcrit; and platelet large cell ratio.

### Flow cytometry analysis of the immune cells (PBMCs and PMNs) of dairy cows

The PBMCs and PMNs were isolated from dairy cows under normothermic and hyperthermic conditions. For analyses, PBMCs and PMNs from 6 Holstein and 8 Jersey cattle were used for each time slot. The isolated PBMCs and PMNs were subjected to flow cytometry (FACS Canto II; BD Bioscience, Heidelberg, Germany) analysis for immune cell population quantification using FlowJo software v10.7.1 (Tree Star Inc., OR, USA). The samples used for surface staining were fixed with 4% paraformaldehyde for 20 min at 4°C, and those used for intracellular staining were fixed with the Perm buffer at 4°C. The isolated cells were stained using a live/dead fixable aqua dead cell stain kit (Invitrogen, CA, USA). Samples were stained with the following direct fluorescence-conjugated antibodies: anti-CD4:Alexa Flour 647 (Bio-Rad, MCA1653A647), anti-CD21:PE (Bio-Rad, MCA1424PE), anti-MHCII:FITC (Bio-Rad, MCA5656F), anti-CD8:Alexa Fluor 647 (Bio-Rad, MCA837A647), anti-WC1:FITC (Bio-Rad, MCA838F), anti-CD16:FITC (Bio-Rad, MCA5665F), anti-CD14:PE (Bio-Rad, MCA1568PE), anti-CD172a:Pe-Cy5 (Bio-Rad, MCA2041C), anti-CH138a (Kingfisher Biotech, WSC0608B-100), and anti-IgM:Alexa Flour 647 (Invitrogen, A21238). All antibodies were diluted to 1:100 in PBS, and analyses were conducted using the following four panels: (1) CD4 and CD21 for CD4 T cells and CD21 and MHCII for B cells; (2) WC1 and CD8 for γδ T and CD8 T cells; (3) CD172a, CD14, and CD16 for monocytes; and (4) CH138a for neutrophils.

### qRT-PCR

Total RNA was isolated from cell samples to examine the respective responses in Holstein and Jersey steers subjected to normothermia and hyperthermia. Samples in Trizol were incubated for 5 min at room temperature, after which 200 μL of chloroform was added to 1 mL of Trizol. The samples were then vortexed for 10 s and incubated for a further 2 min at room temperature for phase separation. Thereafter, the samples were centrifuged at 10,000×*g* for 20 min at 4°C. The resulting upper aqueous phase was transferred into fresh tubes, to which 0.5–1 mL of isopropyl alcohol was added, followed by gentle mixing by shaking. Subsequent to incubation for 10 min at room temperature and centrifugation at 10,000×*g* for 10 min at 4°C, the supernatant was removed, and the resulting RNA pellet was washed with 75% ethanol prior to being stored in DEPC water (Invitrogen, CA, USA). cDNA was synthesized using AccuPower RT PreMix (Bioneer, Daejeon, Korea). qRT-PCR was performed using a QuantStudio1 Real-Time PCR system (Applied Biosystems, CA, USA) and Solog^TM^ h-Taq DNA Polymerase (SolGent, Daejeon, Korea). The reaction conditions were as follows: 50°C for 10 min, 95°C for 5 min, 95°C for 15s, and 60°C for 30 s (40 cycles), followed by melting curve analysis. The sequences of the primer sets used for amplification are listed in Table 1.

**Table 1:** List of the primer sequences used for quantitative reverse transcription-PCR

Genes	Primer	Sequences
β-actin	Forward	5′-AGCAAGCAGGAGTACGATGAGT-3′
	Reverse	5′-ATCCAACCGACTGCTGTCA-3′
IL-1β	Forward	5′-TGACCTGAGGAGCATCCTTT-3′
	Reverse	5′-AGAGGAGGTGGAGAGCCTTC-3′
IL-2	Forward	5′-ACCTCAAGCTCTCCAGGATG-3′
	Reverse	5′-CTCTGGGGTTCAGGTTTTTG-3′
IL-10	Forward	5′-AGCCTTGTC GGAAATGATCCA-3′
	Reverse	5′-CTCTCTTCACCTTCTCCACCG-3′
IFN-γ	Forward	5′-GATTCAAATTCCGGTGGATG-3′
	Reverse	5′-AAATATTGCAGGCAGGAGGA-3′
IL-18	Forward	5′-TCTGCTCTCCAATGCTTTCA-3′
	Reverse	5′-AGCCATCTTTATGCCTGTGC-3′
IL-17A	Forward	5′-TGAGTCTGGTGGCTCTTGTG-3′
	Reverse	5′-GGTGGAGCGCTTGTGATAAT-3′
TNF-α	Forward	5′-CGGTGG TGGGAC TCGTATG-3′
	Reverse	5′-CTGGTT GTCTTC CAGCTTCACA-3′
IL-6	Forward	5′-CAGCTATGAACTCCCGCTTC-3′
	Reverse	5′-CGGTTTTCTCTGGAGTGGTC-3′
IL-4	Forward	5′-TTGCTGCCCCAAAGAACACAA-3′
	Reverse	5′-TGCTCGTCTTGGCTTCATTCA-3′
MPO	Forward	5′-TACCAGACGCCCAACAACATT-3′
	Reverse	5′-TTCTTGCTGAACACGCCCTT-3′
Hsp70	Forward	5′-AACATGAAGAGCGCCGTGGAGG-3′
	Reverse	5′-GTTACACACCTGCTCCAGCTCC-3′
Hsp90	Forward	5′-GGAGGATCACTTGGCTGTCA-3′
	Reverse	5′-GGGATTAGCTCCTCGCAGTT-3′

### Statistical analysis

Statistical analyses of dairy cow respiration rate and rectal temperature were conducted using the Prism software (GraphPad, La Jolla, CA, USA). One-way ANOVA with Tukey’s post hoc test and two-way ANOVA with Bonferroni post hoc tests were used to calculate statistical significance. All data are presented as mean ± SD, and significance was evaluated as P < 0.05, P < 0.01, and P < 0.001.

## Results

### Changes in hematological parameters in Holstein and Jersey steers under different THI conditions

**Fig. 1:**
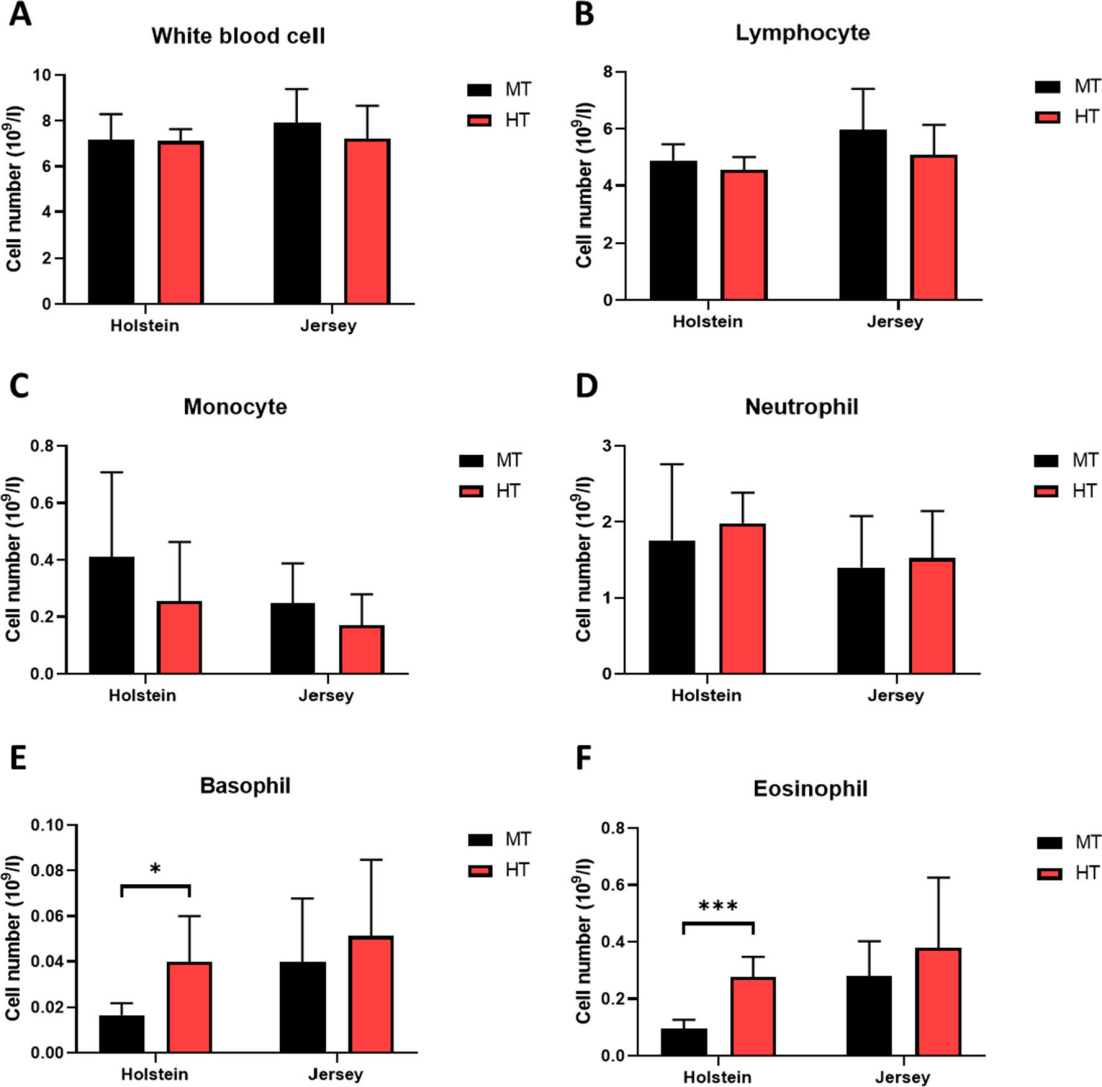
Environmental stress-induced shifts in the concentrations of blood immune cells of Holstein and Jersey cattle. Changes in the blood immune cell profiles of Holstein and Jersey steers subjected to heat stress were assessed by complete blood count (CBC) analysis. CBC measurements showing total counts of **A** white blood cells (WBCs), **B** lymphocytes, **C** monocytes, **D** neutrophils, **E** basophils, and **F** eosinophils. * = P < 0.05, *** = P < 0.001. MT indicates moderate THI condition and HT indicates high THI condition. Error bars denote standard derivations (SD)

To determine whether heat stress alters hematological parameters in steers, we analyzed the whole blood of Holstein and Jersey steers using a Vetscan® HM5 hematological analyzer, which provides a fully automated report for a 22-parameter complete blood count (CBC) from whole blood, discriminating and quantifying cell numbers based on cell size. Whole blood was obtained from Holstein and Jersey steers under two different environmental conditions (MT, average THI = 64.92 and HT, average THI = 79.13). The most prevalent white blood cell populations (lymphocytes, neutrophils, monocytes, basophils, and eosinophils) were enumerated (10^9^ cells/L) and reported as a percentage of total white blood cells (WBC). The results indicated that the levels of total WBCs were not significantly altered by the different THI conditions. Similarly, for both cattle breeds, we detected no significant changes in the cell count and frequency readings obtained under each environmental condition (P > 0.9) (Fig. 1A). Furthermore, for both Holstein and Jersey steers, there were no significant differences between the MT and HT conditions with respect to the number of lymphocytes, the most abundant type of immune cells in the blood (>10^9^ cells/L) (Fig. 1B). However, although the number of monocytes tended to decline in response to heat stress, the difference was not significant (Fig. 1C). In addition, the number of neutrophils was non-significantly affected by environmental conditions (Fig. 1D). Similarly, we detected no significant differences in the WBC, lymphocyte, monocyte, or neutrophil counts between Holstein and Jersey steers (Fig. 1A–D). In contrast, we detected several differences with respect to the number of basophils and eosinophils. In Holstein steers, we observed significant increases in the number of basophils and eosinophils under HT conditions (Fig. 1 E and F); contrastingly, although the number of basophils and eosinophils in Jersey steers was slightly higher under HT condition, it was not significantly different from that observed in Holstein steers. In this regard, it should be noted that there appeared to be an intrinsic breed difference with respect to blood basophils and eosinophils, with Holstein steers having significantly higher numbers of basophils and eosinophils under MT conditions. Collectively, these findings indicate that among the immune-related cells in blood, basophils and eosinophils are sensitive to heat stress, and that these immune cell responses are more prominent in Holstein steers.

### Changes in the composition of monocyte subsets in Holstein and Jersey steers under different THI conditions

**Fig. 2:**
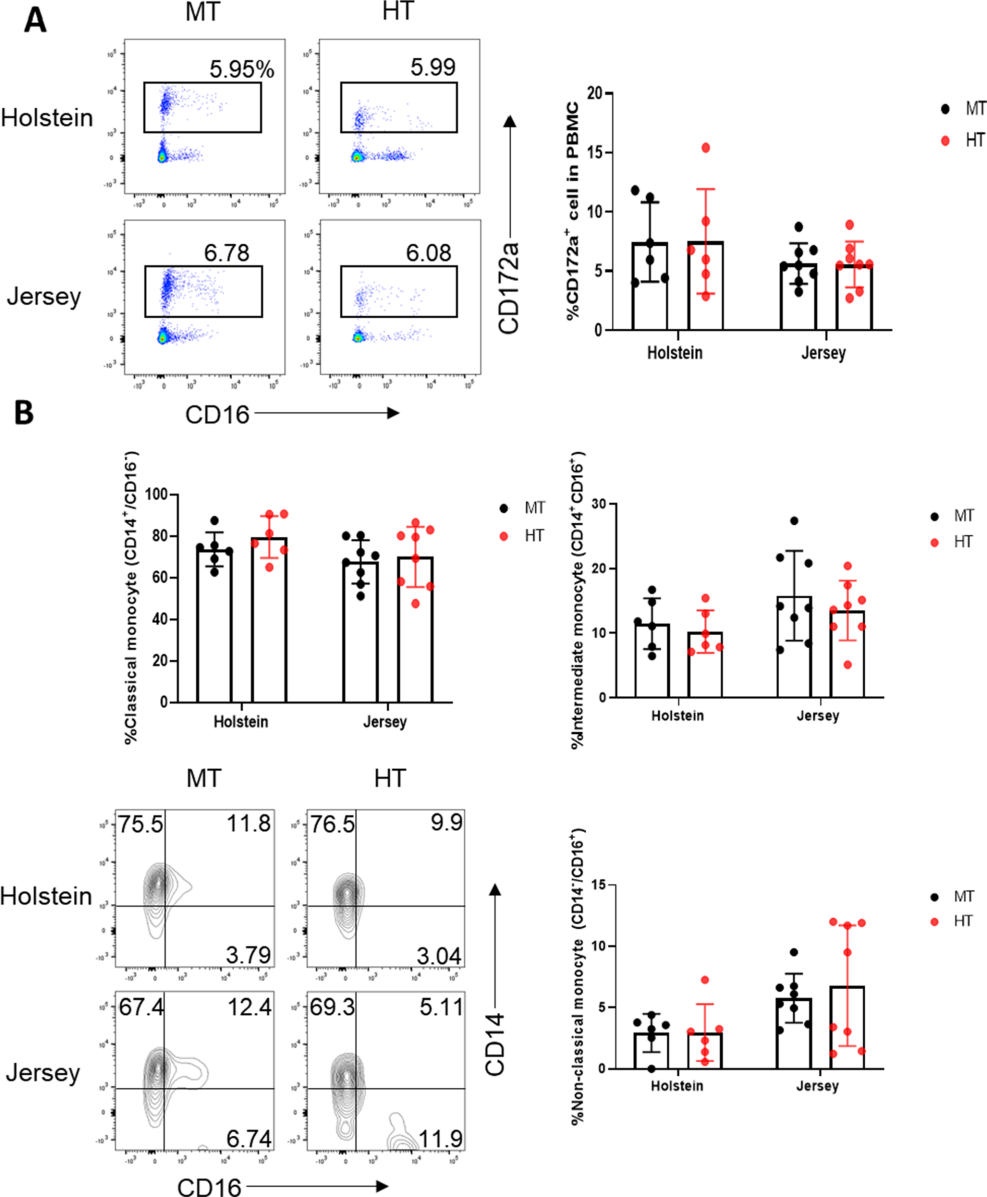
Changes in the monocyte subsets of PBMCs in Holstein and Jersey steers subjected to heat stress. Flow cytometry analysis to identify monocyte subset population. **A** CD172a^+^ monocyte cells were sorted from total PBMCs and **B** a dot plot depicting the three monocyte subsets in monocytes (CD172a^+^): classical CD14^+^CD16^-^, intermediate CD14^+^CD16^+^, and non-classical CD14^-^CD16^+^ monocytes. MT indicates moderate THI condition and HT indicates high THI condition

We subsequently examined blood immune cell composition comprehensively, based on flow cytometric analyses, by initially analyzing the innate immune cells, monocytes, and their subsets. Among viable PBMCs, monocytes were identified as CD172^+^ cells (Fig. 2A). In total PBMCs, we detected 5 to 6% monocytes, the proportions of which did not differ significantly between Holstein and Jersey steers. Similarly, environmental conditions (MT vs. HT) appeared to have no significant effect on the proportion of monocytes. Furthermore, among total monocytes, we identified three subsets based on expression of the surface markers CD14 and CD16 (Fig. 2B). Bovine monocytes have been classified into classical (CD14^+^CD16^-^), intermediate (CD14^+^CD16^+^), and non-classical (CD14^-^CD16^+^) types. Consistent with the findings of a previous study, we found that the classical type constituted the majority (89%) of observed bovine monocytes, with the intermediate and non-classical monocytes being present in minor proportions (5–10% for each subset) (Hussen et al. 2013; Hussen and Schuberth 2017). However, for both the cattle breeds, we detected no significant differences in the populations of monocyte subsets under MT and HT conditions.

### Changes in composition T and B-lymphocytes in PBMCs in response to heat stress

**Fig. 3:**
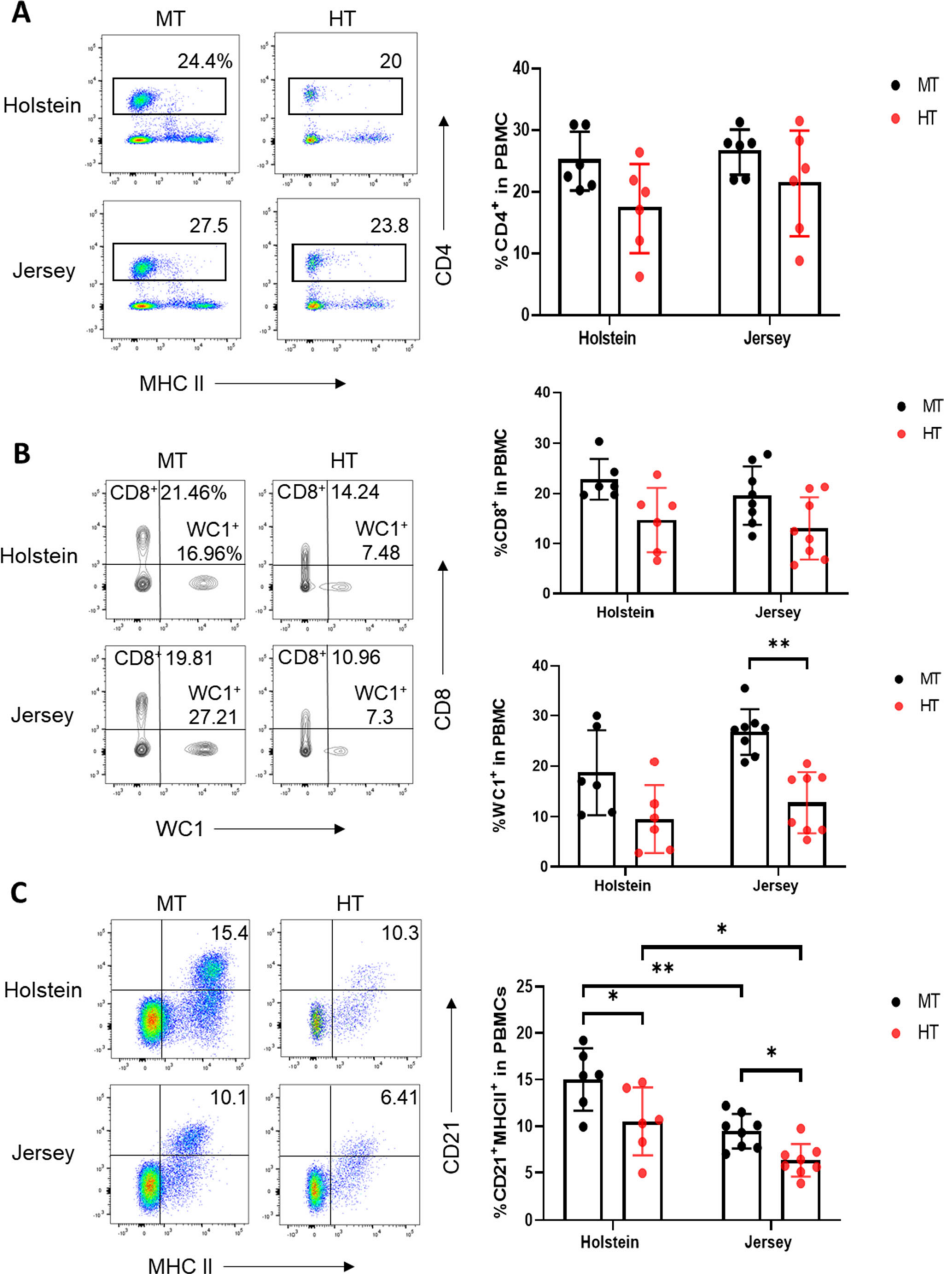
Changes in T and B lymphocytes among PBMCs in Holstein and Jersey steers subjected to heat stress. Flow cytometry analysis to identify lymphocytes subset population. Heat stress caused reduction in lymphocyte populations in the PBMCs of both Holstein and Jersey steers. The lymphocytes comprised **A** CD4^+^ T cells, **B** CD8^+^ T cells and WC1^+^ γδ T cells, and **C** CD21^+^MHCII^+^ B cells. MT indicates moderate THI condition and HT indicates high THI condition. * = P < 0.05, ** = P < 0.01

To elucidate the immunological status of lymphocytes under HT conditions, we evaluated the diverse types of T cells in PBMCs, based on an assessment of the different T cell subsets, namely T helper cells expressing CD4^+^ (Fig. 3A), T cytotoxic cells expressing CD8^+^ (Fig. 3B), and γδ T cells expressing WC1^+^ (Fig. 3B), in heat-stressed dairy cows. No significant differences were detected with respect to the frequency of CD4^+^ T cells. We observed decrease in the number of both CD8^+^ and WC1^+^ T cells in both Holstein and Jersey steers under HT conditions. However, while there was no significant difference the number of CD8^+^ T cells in Jersey steers, the proportion of WC1^+^ T cells was found to be significantly lower under HT conditions than under MT conditions. Taken together, these findings indicate that heat stress conditions induce changes in the T-lymphocyte composition of PBMCs, particularly WC1^+^ T cells, in Jersey steers.

To identify bovine B cells, we stained PBMCs immunologically with the B cell markers CD21 and MHCII and observed that B cells were gated on the CD21^+^MHCII^+^ B cells in PBMCs (Fig. 3C). Interestingly, we observed significant differences and changes in the CD21^+^MHCII^+^ B cell population, with significant reduction in the proportion of B cells being detected in the steers of both breeds under HT environmental conditions (Fig. 3C). Moreover, we observed differences in the B cell proportions between Holstein and Jersey steers under MT conditions, with Jersey steers having a significantly lower proportion of CD21^+^MHCII^+^ B cells than Holstein steers. Collectively, these finding indicated that heat stress environmental conditions reduce B cell counts in both Holstein and Jersey steers and that there are differences between the breeds in this regard.

### Change of mRNA expression for cytokine in PBMCs by heat stress environment

**Fig. 4:**
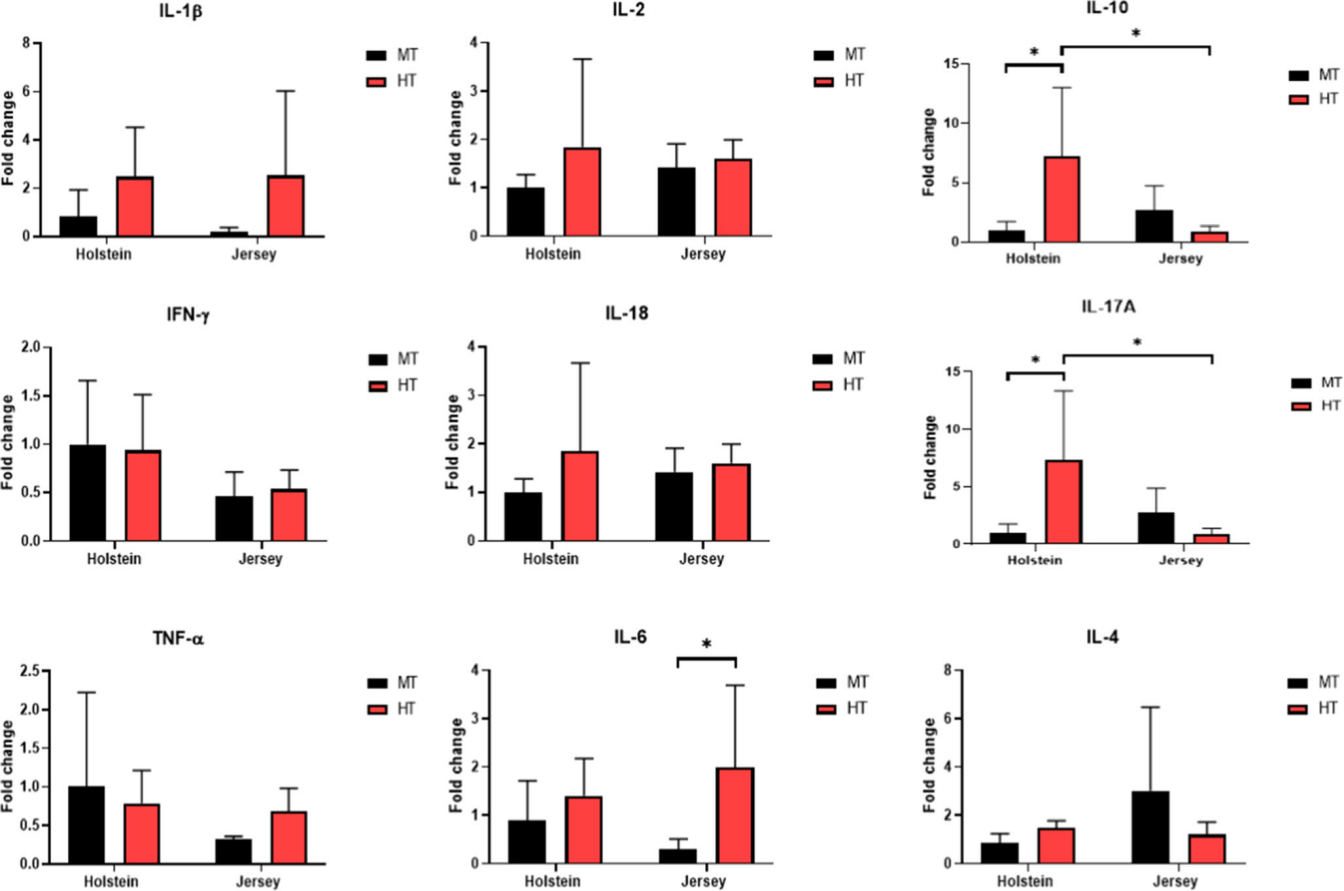
Changes in the mRNA expression of cytokines in the PBMCs of Holstein and Jersey steers subjected to heat stress. The expression of IL-1β, IL-2, IL-10, IFN-g, IL-18, IL-17A, TNF-a, IL-6, and IL-4 was examined in PBMCs using qRT-PCR. MT indicates moderate THI condition and HT indicates high THI condition. Statistical significance is denoted by an asterisk (*). * = P < 0.05

To examine the functional changes in PBMCs induced by heat stress, we assessed the expression of cytokine mRNAs in PBMCs based on real-time PCR analysis of the expression of IL-1β, IL-2, IL-10, IFN-γ, IL-18, IL-17A, TNF-α, IL-6, and IL-4 in the PBMCs isolated from Holstein and Jersey steers. The results revealed significant increase in the mRNA expression of IL-10 and IL-17A in Holstein steers in response to temperature alleviation (P < 0.05) (Fig. 4). In addition, the mean levels of IL-10 and IL-17A mRNA expression in Holstein steers were significantly higher than those in Jersey steers under HT conditions (P < 0.05), as determined by two-way ANOVA with Bonferroni correction. Furthermore, we detected a significant increase in the expression of IL-6 mRNA in Jersey steers under HT conditions (P < 0.05), although no significant changes in expression were observed in Holstein steers. However, no significant differences were observed in the levels of IL-1β, IL-2, IFN-γ, IL-18, TNF-α, IL-6, and IL-4 (Fig. 4) under heat stress or between the two breeds.

### Changes in neutrophils by heat stress environment

**Fig. 5:**
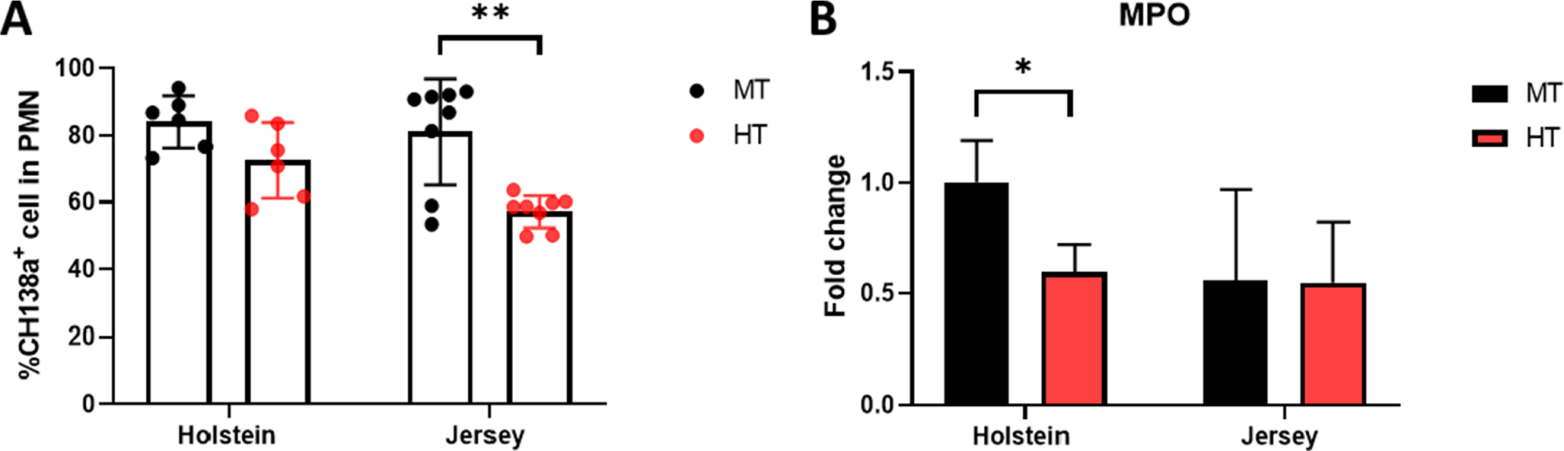
Altered neutrophil responses in heat-stressed Holstein and Jersey steers. **A** A graph showing the CH138a ^+^ cell population in PMNs. **B** mRNA expression of MPO in PMNs under different environmental conditions. MT indicates moderate THI condition and HT indicates high THI condition. Significant differences are denoted by asterisks (*). * = P < 0.05, ** = P < 0.01

We subsequently sought to determine whether heat stress induces changes in blood neutrophils based on an analysis of isolated PMNs. Neutrophils are among the most abundant immune cell types in the blood and can be identified based on the expression of CH138a (Piepers et al. 2009; Della Libera et al., 2015). We accordingly found that the population of CH138a^+^ cells in PMNs underwent a decline in the steers exposed to HT conditions (Fig. 5A); this change was more evident in Jersey steers, which showed a significantly reduced proportion of neutrophils. In addition, we detected a significant reduction in the expression of neutrophil protein myeloperoxidase (MPO) in Holstein steers only under HT conditions (Fig. 5B). These observations indicate that heat stress has a significant effect on neutrophils (number and function) in the blood of steers.

### Changes in mRNA expression for heat shock proteins in PBMCs by heat stress environment

**Fig. 6:**
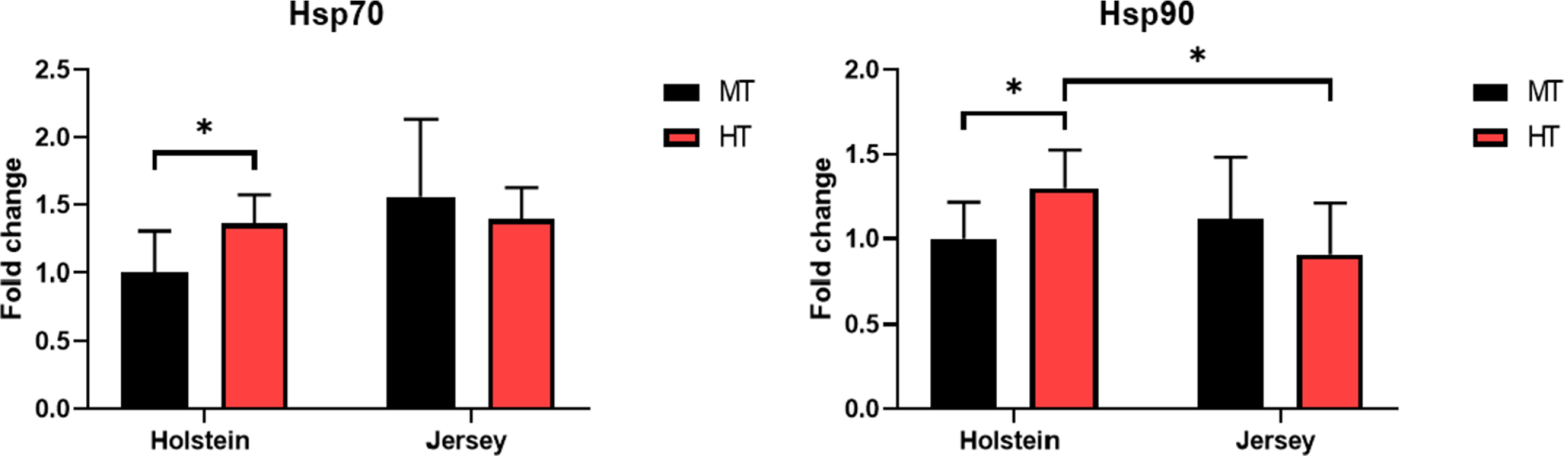
Changes of mRNA expression for heat shock proteins in PBMCs in heat-stressed Holstein and Jersey steers. The expression of Hsp70 and Hsp90 was examined in PBMCs by using qRT-PCR. Significant differences are denoted by an asterisk (*). * = P < 0.05

Heat shock proteins have been served as crucial biomarkers in response to stress tolerance and adaptation (Hassan et al. 2019). Additionally, we have examined the expression of heat shock proteins (Hsp) genes in PBMCs of Holstein and Jersey steers between MT and HT condition. Interestingly, the expressions of Hsp70 and Hsp90 in PBMCs isolated from Holstein steers were significantly increase under heat stress environment (P < 0.05) (Fig. 6). But, there was no significant difference in the expression of Hsp70 and Hsp90 of Jersey steers. This leads to significantly higher mRNA expression of Hsp90 in PBMCs from Holstein steers than those in Jersey steers in HT condition.

## Discussion

The combination of high temperature and humidity creates a hostile environment that can readily overwhelm the capacity of cattle to dissipate heat load, leading to increases in body temperature that may exceed physiological limits (Ronchi et al. 1997). As in other mammals, livestock generally maintain body temperature within a suitable range under certain environmental conditions, referred to as the thermoneutral zone. This zone causes minimal heat generation at normal rectal temperatures, within which the maximum production efficiency is achieved under minimal physiological expenditure. At ambient temperatures above 26°C, cows are prone to heat stress, a condition in which it becomes difficult for these animals to control their body temperature (Kadzere et al. 2002). Chronic heat stress can cause persistent dysfunction of the hypothalamus–pituitary–adrenal axis, which induces physiological changes, including metabolic disorders, immunosuppression, increased vulnerability to diseases, and reproductive disorders (Narayan and Parisella 2017; Ju et al. 2014).

It is generally accepted that heat-stressed animals are unhealthy and susceptible to various diseases. For example, animals exposed to heat stress have been identified as clinically vulnerable to heat stroke, bovine respiratory disease, rumen acidosis, and mastitis (Louie et al. 2018; Zhao et al. 2019; Vitali et al. 2020). However, a majority of the studies in this field have focused primarily on the physiological changes and nutritional perspectives associated with high-temperature stress; there have been comparatively few studies that have assessed the immunological implications. Nevertheless, attention is increasingly being devoted to gaining an understanding of the heat stress-associated mechanisms underlying changes in immune system function and the increased susceptibility of dairy cows to disease. Among domestic animals, high-productivity dairy cows have been identified as the most sensitive to heat stress. In this regard, however, beef cattle are considered less important than dairy cattle, given that beef cattle generally have a higher average body temperature, which is associated with a lower metabolic rate and the production of less body heat that needs to be dissipated in order to avoid heat stress (St-Pierre et al. 2003; Nardone et al. 2010). Despite this apparent advantage, however, beef cattle remain particularly vulnerable to extreme environmental conditions and, thus, need to compensate for their higher body temperature through homeostatic mechanisms such as panting, sweating, and urination. It is relatively easy to identify behavioral changes associated with heat stress. For example, heat-stressed beef cattle can be characterized by enhanced water intake and reduced feed intake and activity. However, little information is currently available regarding the altered immune function of steers under hyperthermal conditions. Thus, in the present study, we assessed changes in the composition and function of different types of blood immune cells in steers under normal and heat stress conditions.

Blood comprises multiple types of cells including granulocytes, a type of leukocytes, which are active in the front-line defense against pathogen invasion and can be divided into three main subsets, namely, neutrophils, eosinophils, and basophils. Given their versatility, these cells make a substantial contribution to the activation of adaptive immune responses (Stone et al. 2010; Scapini and Cassatella 2014). Both basophils and eosinophils are associated with allergic inflammation and have been considered primary effector cells against parasitic infection and allergen. The basophils initially accumulate in sites at which inflammation induces hypersensitivity reactions during immune responses designed to counteract the activity of allergens and parasite through regulation of Th2 reactions. Eosinophils, whose recruitment is related to the secretion of chemokines, cytokines, interleukins, and other products such as histamine (Fulkerson and Rothenberg 2013; Nadif et al. 2013), are associated with type 2 immune response as well. Interestingly, on the basis of our CBC analyses, basophil and eosinophil numbers were numerically increased in response to heat stress. It has been reported that eosinophils and basophils are responsive immune cells to heat stress, but they show differential responses. Consistently, several studies have reported that animals have increased basophil counts following exposure to heat stress (Mitchell et al. 1992; Maxwell et al. 1992; Altan et al. 2000). The occurrence of basophilia, an abnormal increase in basophil levels under conditions of extreme stress, is generally accepted as a reliable indicator of stress, disease severity, and chronic inflammation and infections (Valent et al. 2018; Feriel et al. 2020; Mitchell et al. 1992; Altan et al. 2000; Maxwell 1993; Maxwell and Robertson 1998). However, eosinophils respond differently to heat stress, and in this regard, it has been documented that there is a reduction in the eosinophil counts of calves exposed to high temperatures (da Silva et al. 1992; Alhidary et al. 2012). As a part of type 2 immunity, the basophil numbers in blood and mesenteric lymph nodes expanded during parasite infection (Roland et al. 2014; Reitz et al. 2018). In addition, eosinophilia has been shown to be a reliable diagnostic clue for a helminth and bacterial infections (Ramirez et al. 2018). Relevant studies conducted on the influence of heat stress on host immune homeostasis have been fully elucidated that exposed to heat stress consequently increased the risk of external parasites and vector-borne diseases (Patz et al. 2000). On the basis of cellular and biological aspects of basophil and eosinophil, there is a higher chance that several parasitic infections could be happened under heat stress environment. Therefore, we could not rule out the possibility that heat-stressed steers suffered parasitic infection during HT condition in this study.

The findings of the present study indicate that basophil and eosinophil responses may differ between Holstein and Jersey steers, with Jersey steers having higher basophil and eosinophils levels than the Holstein steers under MT conditions. Accordingly, this could be considered a breed-specific immunological phenotype; in line with this observation, we found that the basophilic and eosinophilic responses in Holstein steers were more prominent than those detected in Jersey steers. Although the responses of these cells have yet to be sufficiently characterized, the evidence obtained to date indicates that altered eosinophil and basophil responses may be associated with diseases linked to heat stress. When we consider the major function of the immune cells against the parasitic infection and host defense, there are possibilities that heat stress induces the basophil and eosinophil’s over-reaction to the antigens and finally renders the animals more vulnerable to the infections under heat stress environment. Collectively, our findings indicate that the granulocytes basophils and eosinophils are sensitive to heat stress and that the response to this source of stress is more pronounced in Holstein steers. Thus, we suggest that the blood counts of basophils and eosinophils could serve as potential biomarkers for determining heat stress in Holstein steers.

With regard to characterizing the immunological properties of animals, flow cytometry analysis represents a powerful tool in the field of immunology. Surprisingly, however, comparatively few flow cytometry-based studies have been conducted to assess immunological responses in the field of animal science, which accordingly motivated us to use this technique to assess the heat stress-related mechanisms of the blood immune system of Holstein and Jersey steers.

Among PMNs, neutrophils play key roles as the main cellular type involved in eradicating microorganisms and preventing damage to host cells (Henson and Johnston Jr, 1987). Neutrophils bind to and ingest microorganisms via phagocytosis, and the combined activity of neutrophil reactive oxygen species (ROS) and granule constituents, such as MPO, is highly effective in killing most bacteria and fungi (Kobayashi et al. 2005). Accordingly, a substantial reduction in the number of neutrophils or defects in their antimicrobial activity markedly enhances the likelihood of elevated rates of morbidity and mortality in patients with bacterial infections (Nauseef 2007). Moreover, neutrophils serve as an effective marker for assessing the effects of heat stress on the immune system based on determination of the neutrophil-to-lymphocyte ratio, which has been established as an indicator of adverse outcomes in oncology patients, with associations between this ratio and mortality being confirmed in both human and animal studies (Zahorec 2001; Ni et al. 2019). In some cases, in vivo heat stress has been found to be associated with reduction in the levels of neutrophil phagocytosis (Tejaswi et al. 2020). Other studies have reported that inadequate levels of antioxidants and trace elements, which may occur under heat stress conditions, can lead to a reduction in the number of neutrophils in the blood and an increase in the incidence of mastitis and retention of the placenta in dairy cows (Spears and Weiss 2008; Smith et al. 1997). Our flow cytometric results in the present study revealed a reduction in the percentage neutrophil composition of PMNs in Holstein steers under HT conditions, which was significantly lower than that recorded in Jersey steers (Fig. 5A). However, our CBC observations indicated that there were no significant differences in neutrophil numbers in the whole blood of the two breeds. We suspect that this discrepancy between the findings of CBC and flow cytometry analyses could be attributable to changes in the numbers of other immune cells in the blood, as we observed increases in the numbers of basophils and eosinophils in response to heat stress.

MPO is a cationic heme-containing enzyme found in the primary granules of neutrophils, which is released into both the phagolysosomal compartment and the extracellular environment in response to oxidative stress and during inflammatory responses (Aratani 2018). The paradigm of MPO release from activated neutrophils is normally related to rectifying disease states and combating different types of microbial activities at the sites of infection (Khan et al. 2018). It has been demonstrated that when bovine PMNs are exposed to excessively high temperatures in in vitro culture, they show impaired neutrophil function. Generally, it has been established that a reduction in MPO functionality represents a good predictor of immune suppression (Boulougouris et al. 2019). In the present study, we found that a change in environmental heat stress induced a reduction in the expression of MPO only in Holstein steers (Fig. 5B). Therefore, the impaired function of immune cells, such as attenuated neutrophil MPO expression, could be attributable to thermal stress, the effects of which were more evident in Holstein steers.

PBMCs, which are the prominent type of lymphocytes (70–90%), are essential mediators of cell-mediated and humoral immune responses; functions of PBMCs on the immune system are typically investigated by measuring changes in cytokine secretion, proliferation, or gene expression (Akdis et al. 2012; Crotty 2011; Kleiveland 2015). Among innate immune cells, γδ T cells expressing WC1 are associated with the production of pro-inflammatory cytokines and they directly attack target cells, such as infected cells, through their cytotoxic activity, or via the activation of other types of immune cells (Rogers et al. 2005; Lawand et al. 2017). Relevant studies have demonstrated that γδ T cells are the most sensitive to environmental stress. For example, the effect of weaning in beef calves has been found to be associated with a reduction in peripheral lymphocyte count and impaired phagocytic function, with transient reduction in the proportion of γδ T cells (Lynch et al. 2010). In addition, the administration of dexamethasone has been demonstrated to induce a disproportionate depletion of γδ T cells, which results in a substantial suppression of gene transcription in lymphocytes (Menge and Dean-Nystrom 2008). Here, we found that stress-induced environmental changes had a substantial influence on WC1^+^ lymphocytes, which is in line with the findings of previous studies that have observed adverse environmental effects, including transportation stress, parturition, and dexamethasone treatment, in bovines (Riondato et al. 2008; Meglia et al. 2005; Menge and Dean-Nystrom 2008). In the present study, we also observed that heat-stressed Holstein and Jersey steers showed numerical reduction in the WC1^+^ lymphocyte populations (Fig. 3B), which indicates that γδ T cells are innate immune cell types that are sensitive to heat stress in steers and that these changes may be associated with a reduction in cell-mediated immunity.

The most evident heat stress-related change detected in this study was a reduction in the number of blood CD21^+^MHCII^+^ B cells in the steers of both the breeds (Fig. 3C). B cells differentiate into plasma cells that produce antibodies and memory cells. B lymphocytes not only play essential roles in the production of antibodies but also serve as a source of cytokines. Stress-related changes in B lymphocytes have also been documented. For example, exposure to heat stress has been demonstrated to promote reduction in B lymphocyte differentiation and replication in numerous species, including rats, birds, and cows (Pitkin 1965; Regnier and Kelley 1981; Kelley et al. 1982). In addition, heat stress results in reduction in the levels of immunoglobulins (IgA, IgM, and IgG) and cytokines, ultimately causing immunosuppression (Guy et al. 2017; Safa et al. 2019). Other studies have shown reduced passive immunity under ambient temperature, with lower levels of circulating IgG than those under MT conditions (Machado-Neto et al. 1987). In addition, it has been reported that heat stress negatively affects immune responses following vaccination. Moreover, antibody levels and antibody titers in the Newcastle disease have been reported to decline in response to heat stress (Zulkifli et al. 2000; Liew et al. 2003), whereas chronic heat stress induces reactions that inhibit the production of IgG and IgG1 (Hu et al. 2007). These observations are consistent with the reduced numbers of B cells under heat stress conditions detected in the present study. Thus, we believe that a better understanding of the immunosuppression of B cell immunity-induced risk factors under HT conditions is necessary to facilitate alleviation of the detrimental effects of heat stress on steers.

The findings of previous studies have indicated that under the conditions of heat stress, there is an increase in the expression of chaperones and heat shock genes that act to prevent protein aggregation and misfolding, which contributes to cell survival and triggers immune system activation, whereas exposure to severe heat stress can lead to the expression of genes involved in apoptotic processes (Srikanth et al. 2017). In the present study, we found that environmental heat stress induced increase in the expression of Hsp genes in PBMCs isolated from Holstein steers alone (Fig. 6). Hsp70 and Hsp90 have major function in cellular thermotolerance, immune modulation, and reliable marker for heat stress (Hassan et al. 2019; Deb et al. 2014). In this regard, the obtained observation strengthens the evidence that there are definite breed-specific differences for PBMC heat stress response between Holstein and Jersey steers. This may be explained by differential susceptibility to heat stress in host homeostasis.

In this study, we observed reduction in the number of several essential immune cell types, including B cells and γδ T cells, in response to heat stress. Lymphocyte counts can serve as an indicator signifying that hosts are mounting appropriate immune protective responses against bacterial and viral antigens. Stress is a primary cause of altered lymphocyte trafficking and function, rendering animals more susceptible to infectious diseases (Lalor and Hepburn 2017); the altered immune cell populations due to heat stress may potentially enhance the susceptibility to pathogenic infection, thereby explaining an increase in the incidence of infectious diseases in animals suffering from heat stress. It is conceivable that different immune cell types are characterized by different sensitivities in their responses to stress. However, at present it is not fully understand how and which factors contribute to the observed reduction in immune cell populations in the blood, or which factors may influence immune cell survival in response to heat stress.

In this regard, relevant studies have focused on the effects of heat stress on the inhibition of immune cell growth and proliferation. However, immune cell development and differentiation can be affected by a diverse range of additional factors, including hormones and metabolites, and several studies have reported that heat-stressed animals are typically characterized by altered metabolic profiles (Ganesan et al. 2018; Malmendal et al. 2006; Min et al. 2017). Given that diverse metabolites can differentially regulate immune cell differentiation and functions, changes in metabolic profiles may, in turn, induce changes in the population of blood immune cells under heat stress conditions. The data presented herein indicate that heat stress alters the immune cell populations (B-lymphocytes and γδ T cells) and function (MPO expression by PMNs) and provide evidence that heat stress-induced immunosuppression can be attributed to abnormal blood immune cell responses. However, we gained a comparatively little insight regarding the function of these immune cells associated with the responses to pathogens or antigens. In this regard, further investigations based on vaccine and/or antigen challenge models that examine antibody titers under heat stress conditions could represent a promising approach to gain a better understanding of the changes in immune cell function. In summary, prolonged heat stress impairs immune function in steers, which is a basis for the development of immune incompetence, elevated disease vulnerability, and the incidence of immune-related diseases in steers. We accordingly believe that it is necessary to develop additional management strategies based on the altered immune responses attributable to heat stress.
